# Identification and characterization of circRNAs involved in the regulation of low nitrogen-promoted root growth in hexaploid wheat

**DOI:** 10.1186/s40659-018-0194-3

**Published:** 2018-11-03

**Authors:** Yongzhe Ren, Huifang Yue, Le Li, Yanhua Xu, Zhiqiang Wang, Zeyu Xin, Tongbao Lin

**Affiliations:** 1 College of Agronomy, Henan Agricultural University, Zhengzhou, 450002 China; 2 State Key Laboratory of Wheat and Maize Crop Science, Henan Agricultural University, Zhengzhou, 450002 China; 3 Collaborative Innovation Center of Henan Grain Crops, Henan Agricultural University, Zhengzhou, 450002 China; 4 Shangqiu Normal University, Shangqiu, 476000 China

**Keywords:** *Triticum aestivum* L., Root, Low nitrogen, CircRNAs

## Abstract

**Background:**

CircRNAs are widespread in plants and play important roles in response to abiotic stresses. Low nitrogen (LN) promotes the growth of plant root system, allowing it to explore more nitrogen. However, whether circRNAs involved in the response to LN stress and the regulation of LN-promoted root growth in wheat remains unclear.

**Methods:**

Two wheat varieties (LH9 and XN979) with contrasting root phenotypes to LN stress were used as materials to identify circRNAs under control and LN conditions by using high-throughput sequencing technology.

**Results:**

Six differentially expressed circRNAs (DECs) involved in the common response to LN stress and 23 DECs involved in the regulation of LN-promoted root growth were successfully identified. GO analysis of the DEC-host genes involved in the regulation of LN-promoted root growth showed that GO terms related to biological regulation, responses to stimuli and signalling were significantly enriched. Moreover, seven DECs were predicted to have miRNA binding sites and may serve as miRNA sponges to capture miRNAs from their target genes.

**Conclusions:**

LN stress altered the expression profiles of circRNAs in wheat. This is the first report of LN stress responsive circRNAs in plants. Our results provided new clues for investigating the functions of circRNAs in response to LN stress and in the regulation of LN-promoted wheat root growth.

**Electronic supplementary material:**

The online version of this article (10.1186/s40659-018-0194-3) contains supplementary material, which is available to authorized users.

## Background

Plant roots have high plasticity in response to varying different environmental conditions [1–5]. The nitrogen deficiency environment promotes the growth of plant root system, allowing it to reach deeper soil layers and explore more nitrogen. Therefore, the ability to develop a deep root system under nitrogen limiting conditions is of vital importance for nitrogen acquisition.

CircRNAs are a type of endogenous non-coding RNAs derived from mRNA precursor back-splicing [6]. The 5′ and 3′ ends in mature circRNAs have been jointed together, forming covalently closed loop structures [7]. The identification, biogenesis and functions of circRNAs have been widely reported in animals, such as human, mouse and *Drosophila* [8–11]. However, the roles of circRNAs in plants have not attracted enough attention [12]. With the development and wide application of high-throughput sequencing technology, circRNAs have been identified in *Arabidopsis* [13–16], soybean [17], rice [13, 18], tomato [19], barley [20], cotton [21], maize [22], wheat [23] and some other plant species in recent years [24, 25]. Ye et al. identified 12,037 and 6012 circRNAs in rice and *Arabidopsis thaliana*, respectively [13]. Lu et al. also reported 2354 circRNAs in *Oryza sativa* [18]. These reports indicate that circRNAs are widespread in plants and may play important roles in the regulation of plant growth and development. Zuo et al. identified 854 circRNAs and found that 163 of them were chilling responsive in tomato [19]. Zhao et al. identified 1041, 1478, 1311 and 499 circRNAs in diploid progenitors of *Gossypium* spp., *G. arboreum* and *G. raimondii*, their interspecies hybrid and allotetraploid *G. hirsutum*, respectively [21]. Wang et al. isolated 88 circRNAs and found that 62 circRNAs were differentially expressed under dehydration stress conditions compared with well-watered control in wheat [23]. Chen et al. [22] found that circRNAs mediated by transposons are associated with transcriptomic and phenotypic variation in maize. In rice, some circRNAs exhibit differential expression under Pi-sufficient and Pi-starvation conditions, suggesting that circRNAs may play a role in response to Pi starvation stress [13]. These studies indicate that circRNAs may also play important roles in response to abiotic stresses in plants. However, whether circRNAs participate in the response of plants to low nitrogen (LN) stress and the process of LN-promoted root growth remains to be elucidated.

To explore this question, circRNAs expression profiles of two elite Chinese wheat varieties with contrasting phenotypes to LN stress were obtained using high-throughput sequencing technology. Differentially expressed circRNAs (DECs) were identified and further validated using real-time PCR technology. Moreover, target miRNAs of DECs were predicted and discussed.

## Methods

### Plant materials

Xinong 979 (XN979) and Luohan 9 (LH9) are two elite Chinese wheat varieties with contrasting root phenotypes to LN stress. Therefore, XN979 and LH9 were selected as materials to explore potential circRNAs involved in the processes of LN stress response and LN-promoted root growth.

### Plant growth conditions and evaluation of root phenotype

Hydroponic culture was used to investigate the root traits and collect root samples. Methods for seed sterilization, germination, and the growth conditions of wheat plants were conducted according to Ren et al. [26]. Plants were randomly placed and grown in a greenhouse with at least six replications each. Germinated XN979 and LH9 seeds with residual endosperm removed were transferred to CK (2.0 mM NO_3_^−^) and LN (0.1 mM NO_3_^−^) nutrient solutions. The nutrient solutions were refreshed every 2 days and its pH was adjusted to 6.0 with dilute HCl and KOH before refreshing. The maximum root length (MRL) and root dry weight (RDW) of XN979 and LH9 were evaluated at 15 days after transfer. The developmental stages of wheat plants were Zadoks growth scale 12 and 13 under LN and CK conditions, respectively [27]. The roots of XN979 and LH9 under CK and LN conditions were snap-frozen in liquid nitrogen and stored at – 80 °C for RNA extraction.

### Libraries construction and sequencing

Total RNA was extracted using Trizol reagent according to the manufacturer’s instructions. The concentration and purity of total RNA were measured with a NanoDrop ND-1000 spectrophotometer (NanoDrop Technologies, Wilmington, DE, USA). Ribosomal RNAs (rRNA) were depleted using the Epicentre Ribo-Zero Gold Kit (Illumina, San Diego, USA) according to the manufacturer’s instructions. The cDNA libraries were constructed using the rRNA-depleted total RNAs as templates according to the protocol of the mRNA-Seq sample preparation kit (Illumina, San Diego, USA). Then the libraries were sequenced on an Illumina Hiseq 2500 platform (Hangzhou Shangyi biotechnology company, Hangzhou, China). Three biological replicates were analyzed to minimize experimental errors and the number of false positives. The 12 samples were named as LH9_CK-1, -2, -3; LH9_LN-1, -2 -3; XN979_CK-1, -2, -3 and XN979_LN-1, -2, -3, respectively.

### Identification of circRNAs

The clean reads were mapped to the reference genome (*Triticum aestivum* TGACv1.0) by the bowtie2 (bowtie2-2.2.2) alignment method. The reads of linear RNAs can be mapped to the reference genome properly, while the reads at the loop-forming ligation sites of circRNAs cannot be directly aligned to the wheat reference genome. The unmapped RNA-seq reads were used to further detect head-to-tail spliced (back-spliced) sequencing reads by find_circ software. The detected reads were filtered to predict circRNAs based on the recommended setting rules (GU/AG flanking the splice sites, clear breakpoint detection; ≤ 2 mismatches in the extension procedure; The length of the circRNA junctions ≤ 100 kb) [28].

### Differential expression analysis of circRNAs

Differential expression analysis of circRNAs under CK and LN conditions was performed using the DEseq R package. CircRNAs with P value ≤ 0.05 along with |log2 (foldchange)| ≥ 1 were defined as DECs [29, 30].

### Bioinformatics analysis

CircRNA-miRNA interactions were predicted by using the psRNATarget software [31]. Gene Ontology (GO) analysis was performed on the DEC-host genes with the GOseq R packages based on the Wallenius non-central hyper-geometric distribution [32]. IBM SPSS statistics 21 software was used to determine the statistical significance of the data.

### Quantitative real-time PCR and data analysis

A set of divergent primers were designed based on the flanking sequences of head-to-tail splicing sites of circRNAs to confirm and quantify the circRNAs predicted in this study (Additional file 1: Table S1). The primers used for the quantitative analysis of the circRNA-host genes were designed using the Primer 5.0 software and listed in Additional file 1: Table S1. The cDNA samples were used as templates and mixed with primers and SYBR Green PCR Real Master Mix (Tiangen, China) for real-time PCR analysis using a CFX96 Real-Time System (Bio-Rad, USA). The temperature procedure was: 95 °C for 5 min followed by 40 cycles of 95 °C for 15 s, 60 °C for 15 s, and 72 °C for 15 s. *TaActin* was used as a reference gene to normalize the expression level of investigated genes. IBM SPSS statistics 21 software was used to determine the statistical significance of the data.

## Results

### Root phenotypes under control and low nitrogen conditions

**Fig. 1:**
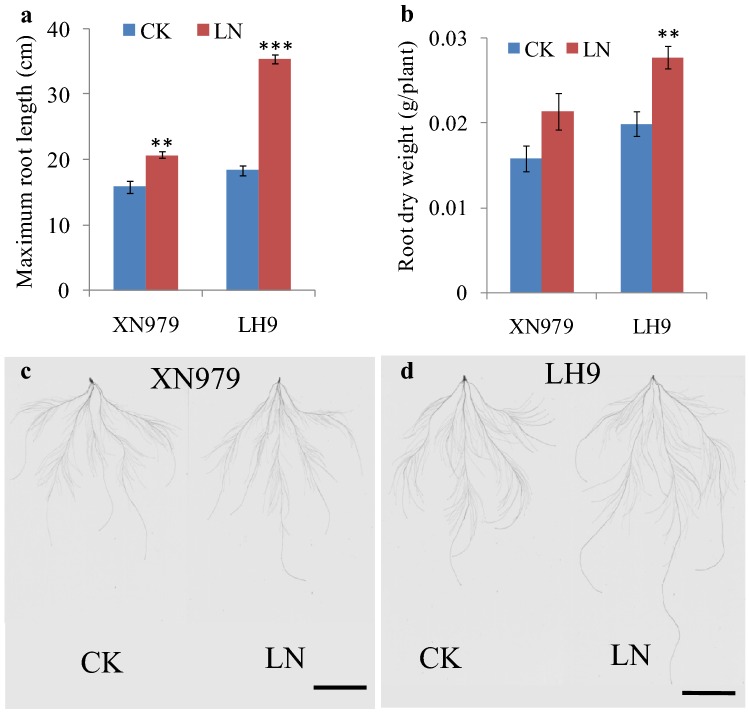
The response of the roots of XN979 and LH9 to low nitrogen (LN) stress. The maximum root length (**a**), root dry weight (**b**) and root morphology (**c**) of XN979 and LH9 under CK (2.0 mM NO_3_^−^) and LN (0.1 mM NO_3_^−^) conditions. Bar = 5 cm

There existed significant genotypic differences of the induction effect between LH9 and XN979. The MRL of XN979 was 4.9 cm longer than control (CK) under LN condition, while the MRL of LH9 was 16.9 cm longer than CK (Fig. 1a–c). Similarly, there was no obvious difference of the RDW of XN979 under LN and CK conditions, while the RDW of LH9 under LN condition was significantly higher than that of CK (Fig. 1b). These results indicated that the root growth of LH9 was more significantly promoted by LN stress than that of XN979.

### CircRNAs identification

**Fig. 2:**
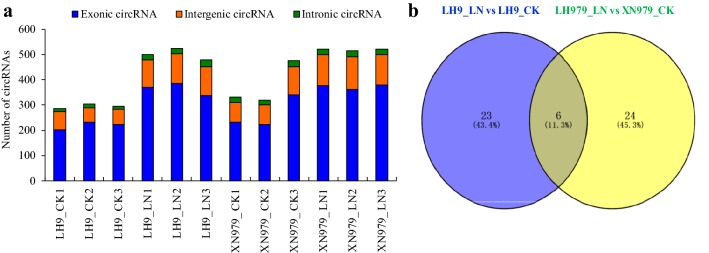
Statistical analysis of circRNAs and differentially expressed circRNAs (DECs) in the roots of LH9 and XN979 under control (CK) and low nitrogen (LN) conditions. **a** The number of exonic circRNAs, intergenic circRNAs, and intronic circRNAs in each sequenced sample. **b** Venn diagram analysis of DECs in the LH9_LN-LH9_CK and XN979_LN-XN979_CK comparisons

To determine circRNAs involved in the processes of LN response and LN-promoted root growth, cDNA libraries from the roots of LH9 and XN979 under CK and LN conditions were constructed and sequenced. Over 100 million raw reads were generated in each library (among 100.9–101.8 million raw reads in each library). About 80–85% reads were mapped to wheat genome and 15–20% unmapped reads were left for circRNAs prediction using find_circ software. The number of circRNAs identified in each sample ranged from 285 to 522, with over 70% of circRNAs being exonic circRNAs (Fig. 2a). The proportion of intergenic circRNA in each sample was between 18.7 and 25.3%. However, the proportion of intronic circRNAs in each sample was no more than 6.5% (Fig. 2a).

### Identification of differentially expressed circRNAs

In the one to one comparison between LH9_LN and LH9_CK, 29 circRNAs were defined as DECs (P value ≤ 0.05 along with |log_2_ (foldchange)| ≥ 1). Among them, 27 circRNAs were up-regulated and two were down-regulated in LH9_LN compared with LH9_CK (Additional file 2: Table S2). In the XN979_LN-XN979_CK comparison, a total of 30 DECs were identified. Among them, 25 circRNAs exhibited up-regulation and five showed down-regulation in the roots of XN979 under LN conditions compared with CK (Additional file 3: Table S3). Among the DECs identified above, 23 DECs were specifically found in the LH9_LN-LH9_CK comparison, 24 DECs were specifically found in the XN979_LN-XN979_CK comparison, and six were present in both comparisons (Fig. 2b; Table 1). Since the degree of LN-promoted root growth is much more pronounced in LH9 than in XN979, the unique DECs in LH9 are likely to play key roles in LN-promoted root growth.

**Table 1:** List of identified circRNAs involved in the regulation of low nitrogen-promoted root growth

CircRNA ID	Position	Chr	Corresponding miRNAs
CircRNA1023	1A:24,294–46,159	TGACv1_scaffold_019564_1AS	tae-miR9660-5p; tae-miR9657b-5p; tae-miR9773; tae-miR1134; tae-miR1133
CircRNA1120	1B:17,689–34,357	TGACv1_scaffold_031043_1BL	tae-miR1117; tae-miR1131
circRNA1393	2B:33,405–113,403	TGACv1_scaffold_146256_2BS	tae-miR9667-5p; tae-miR9652-5p; tae-miR9773; tae-miR9777; tae-miR9772
CircRNA998	5A:6372–31,156	TGACv1_scaffold_374926_5AL	tae-miR1127b-3p; tae-miR1128; tae-miR1135; tae-miR1137b-5p; tae-miR9653a-3p; tae-miR6197-5p; tae-miR1121; tae-miR5049-3p; tae-miR1133; tae-miR1137a; tae-miR9652-5p; tae-miR9773; tae-miR9655-3p
CircRNA866	5B:48,171–103,123	TGACv1_scaffold_404448_5BL	tae-miR7757-5p; tae-miR9663-5p; tae-miR1134; tae-miR1120a; tae-miR9670-3p; tae-miR9773; tae-miR9655-3p; tae-miR1133; tae-miR530; tae-miR9677a
CircRNA1525	U:31,407–76,069	TGACv1_scaffold_642737_U	tae-miR9772; tae-miR9657b-5p; tae-miR9667-5p
CircRNA362	U:19,588–25,979	TGACv1_scaffold_641443_U	tae-miR9780; tae-miR1134; tae-miR9677b; tae-miR9773
CircRNA1168	1A:1774–5928	TGACv1_scaffold_019820_1AS	–
CircRNA977	2A:17,304–20,041	TGACv1_scaffold_113232_2AS	–
CircRNA1198	2B:82,049–141,309	TGACv1_scaffold_146009_2BS	–
CircRNA907	2B:90,859–146,604	TGACv1_scaffold_129685_2BL	–
CircRNA1150	2D:14,143–17,119	TGACv1_scaffold_178493_2DS	–
CircRNA1272	2D:143,637–155,279	TGACv1_scaffold_158033_2DL	–
CircRNA1539	3D:29,228–32,651	TGACv1_scaffold_249219_3DL	–
CircRNA 347	4A:40,570-50,497	TGACv1_scaffold_289349_4AL	–
CircRNA806	4D:50,956–70,562	TGACv1_scaffold_343287_4DL	–
CircRNA982	4D:50,956–70,635	TGACv1_scaffold_343287_4DL	–
CircRNA765	5A:65,727–70,950	TGACv1_scaffold_375076_5AL	–
CircRNA1360	5B:1241–13,921	TGACv1_scaffold_404200_5BL	–
CircRNA962	6A:77,022–79,083	TGACv1_scaffold_485269_6AS	–
CircRNA1683	6A:77,371–794,321	TGACv1_scaffold_485269_6AS	–
CircRNA1258	5D:36,358–43,165	TGACv1_scaffold_434846_5DL	–
CircRNA910	U:88–71,065	TGACv1_scaffold_641580_U	–

### Real-time PCR analysis of DECs and DEC-host genes

**Fig. 3:**
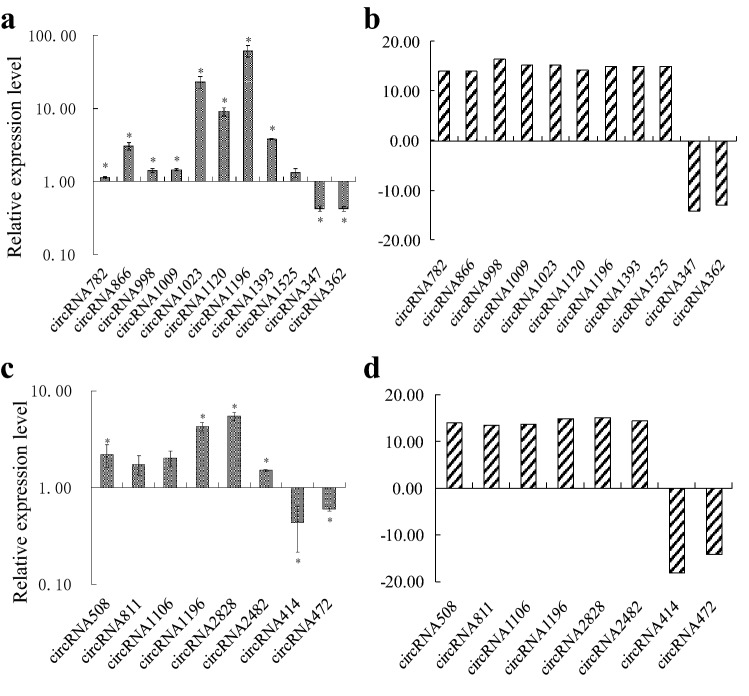
Relative expression analysis of circRNAs under control (CK) and low nitrogen (LN) conditions. **a**, **b** The results of relative expression analysis of LH9 under CK and LN conditions by using real-time PCR (**a**) and RNA-sequence (**b**) technologies, respectively. **c**, **d** The results of relative expression analysis of XN979 under CK and LN conditions by using real-time PCR (**c**) and RNA-sequence (**d**) technologies, respectively; Each bar shows the mean ± standard errors (SE) of three replicates. (*P < 0.05, Duncan’s multiple range test)

**Fig. 4:**
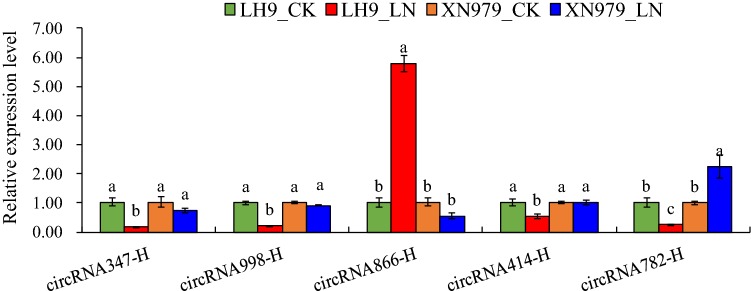
Relative expression analysis of circRNA-host genes in the roots of LH9 and XN979 under control (CK) and low nitrogen (LN) conditions. CircRNA347-H, circRNA998-H, circRNA866-H, circRNA414-H and circRNA782-H represent the host genes of circRNA347, circRNA998, circRNA866, circRNA414 and circRNA782, respectively. Significant difference at < 0.05 is indicated by different letters above the columns

To verify the data of RNA-seq, 11 DECs identified in the LH9_LN-LH9_CK comparison and 8 DECs identified in the XN979_LN-XN979_CK comparison were randomly selected for expression level verification using real-time PCR (Fig. 3). The expression levels of the 29 DECs detected using real-time PCR technology matched well with the results of RNA-seq. This evidence indicated that the results of RNA-seq are reliable. It has been reported that the expression levels of many plant circRNAs showed positive or negative correlations with their DEC-host genes [13, 14]. We selected five DECs including three LH9 unique, one XN979 unique and one common responsive DECs in both varieties to check the expression levels of the DEC-host genes. Results showed that the expression levels of four DECs-host genes showed a positive or negative correlation with their corresponding DECs. However, we did not observe the correlation between the expression levels of circRNA414 and its host gene in XN979 (Fig. 4).

### GO analysis of DECs-host genes of LH9 unique DECs

**Fig. 5:**
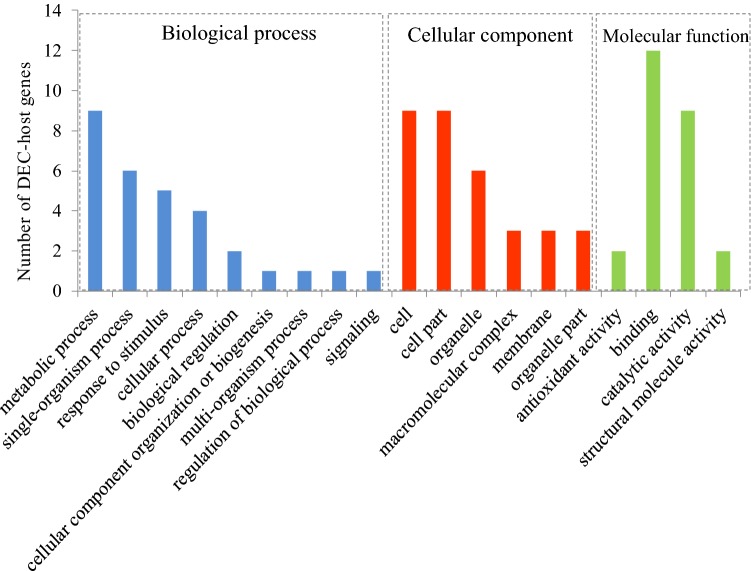
Gene Ontology analysis of the host genes of the differentially expressed circRNAs (DECs) that specifically identified in LH9. Columns in blue color represent GO terms belonging to the biological process category; Columns in red color represents GO terms belonging to the cellular component category; Columns in green color represents GO terms belonging to the molecular function category

To further understand the functions and features of the specifically identified DECs in LH9, the gene ontology (GO) database was adopted to categorize the DECs-host genes. Those DECs-host genes were mainly classified into 19 important functional groups including 9 biological processes, 6 cellular components and 4 molecular functions (Fig. 5). According to the biological process properties, the most abundant term was “metabolic process” with 9 DEC-host genes. GO terms related to biological regulation, responses to stimuli and signalling were also significantly enriched. In the cellular component category, “cell” (9 DEC-host genes) and “cell part” (9 DEC-host genes) were the central categories. The “binding” and “catalytic activity” included 12 and 9 DECs-host genes, respectively, and were the two most dominant terms in molecular function category (Fig. 5).

### The regulation of circRNAs acting as miRNA sponges

Studies have demonstrated that circRNAs could bind miRNAs and act as competing endogenous RNAs of miRNAs [14, 28]. That is, circRNAs could affect gene post-transcriptional regulation by binding to miRNAs and preventing them from regulating their target mRNAs. To detect whether the unique DECs in LH9 can perform similar functions, psRNATarget software was employed to predict potential miRNA binding sites. Ultimately, seven out of the 23 circRNAs were predicted to have two to thirteen corresponding miRNAs binding sites (Table 1).

## Discussion

CircRNAs were once considered to be transcriptional noises in eukaryotes [28]. However, in recent years, they have been proved to be widespread in plants and play important roles in regulating plant growth, development and responding to abiotic stresses [13–25]. Next-generation sequencing technology combined with bioinformatics methods allows us to genome-wide explore circRNAs. Low nitrogen availability limits plant shoot growth, while it promotes the growth of primary and lateral roots, enabling the root system to reach deeper layers of the soil [1, 3, 33]. In wheat, the growth of roots is also promoted by LN stress, but there are significant genotypic effects [34]. In this study, LN stress significantly increased the RDW and MRL of wheat cultivar LH9, but the effects on XN979 were much smaller (Fig. 1). Here, an integrated comparative root transcriptome study of LH9 and XN979 was conducted under CK and LN conditions to explore LN stress responsive circRNAs in hexaploid wheat. Hundreds of circRNAs were identified in each sample. Most of them belong to exonic circRNAs, followed by intergenic circRNAs, and the lowest proportion is intronic circRNAs (no more than 6.5%) (Fig. 2a). The proportion of intronic circRNAs is similar to the results of other reports in *Arabidopsis* (3.8% intronic circRNAs) and tomato (3.6%) [16, 19]. Like other stresses, such as heat shock in *Arabidopsis* [14], chilling in tomato and dehydration in wheat [19, 23], LN stress also altered the expression profiles of circRNA in wheat. In total, we identified 29 and 30 LN-responsive circRNAs in LH9 and XN979, respectively, of which six co-existed in both varieties (Fig. 2b). There were 23 DECs specifically existed in the LH9_LN-LH9_CK comparison (Fig. 2b). As has been mentioned above, the degree of LN-promoted root growth in LH9 is much more pronounced than that of XN979. Therefore, the six and 23 DECs may involve in the processes of common response to LN stress and LN-promoted root growth, respectively. It has been reported that the expression levels of many plant circRNAs showed positive or negative correlations with their host genes [13, 14]. Pan et al. reported that about 70% of 439 circRNAs were expressed in a similar pattern with their host genes after heat shock in *Arabidopsis* [14]. Our results also showed that the expression levels of four out of five DECs-host genes showed a positive or negative correlation with their corresponding circRNAs (Fig. 4). The result is basically consistent with previous study [14]. However, the identified DEC-host genes in this study are still functionally unknown. To understand further the functions of the unique DECs, GO analysis was performed to annotate the biological functions of the DEC-host genes in LH9. Some important terms related to biological regulation, responses to stimuli and signalling were significantly enriched (Fig. 5). These processes may participate in the regulation of LN-promoted root growth.

Although the mechanism of how plant circRNAs function remains to be elucidated, studies in animal and human have confirmed that circRNAs can act as miRNA sponges, trapping miRNAs from its target genes via the ceRNA network [35, 36]. To uncover whether circRNAs in wheat could target miRNAs and involve in the post-transcriptional regulation of genes, psRNATarget software was used to identify potential miRNAs binding sites of the DECs. Seven of the 23 LH9-specific DECs had putative miRNA-binding sites (Table 1). Moreover, all the seven DECs had 2–13 miRNA-binding sites, which was similar with previous reports in human and another study in wheat [23, 26], but was significantly higher than that reported in rice and tomato [18, 19]. Interestingly, some putative circRNA-binding miRNAs have been reported that involved in the response to low nitrogen and other abiotic stresses [37, 38]. For example, miR530 is differentially expressed in response to low nitrogen stress in rice, suggesting that it may play an important role in plant nitrogen utilization [37]. Another miRNA, miR1120a, has been reported that involved in salt stess response [38]. However, none of them have been reported involved in the regulation of LN-promoted root growth. Therefore, although circRNA-mediated post-transcriptional regulation might play an important role in the regulation of LN-promoted root growth, there is still a lot of work to be done to clarify how it plays the regulatory role.

## Conclusions

LN stress altered the expression profiles of circRNAs in wheat. We totally identified six circRNAs involved in the common response to LN stress and 23 circRNAs involved in the regulation of LN-promoted root growth. Some of them might act as miRNA sponges, trapping miRNAs from its target genes via the ceRNA network. To our knowledge, this is the first report of the identification of LN responsive circRNAs in plants. Our results provided clues for investigating the functions of circRNAs in response to LN stress and the regulation of LN-promoted root growth in wheat.

## Additional files

**Additional file 1: Table S1.** Primers used for real-time PCR.

**Additional file 2: Table S2.** List of differentially expressed circRNAs (DECs) in the LH9_LN-LH9_CK comparison.

**Additional file 3: Table S3.** List of differentially expressed circRNAs (DECs) in the XN979_LN-XN979_CK comparison.

## Abbreviations

LN: low nitrogen
DECs: differentially expressed circRNAs
rRNA: ribosomal RNAs
GO: gene ontology

## References

[1] Linkohr BI, Williamson LC, Fitter AH, Leyser HM (2002). Nitrate and phosphate availability and distribution have different effects on root system architecture of *Arabidopsis*. Plant J.

[2] Hochholdinger F, Tuberosa R (2009). Genetic and genomic dissection of maize root development and architecture. Curr Opin Plant Biol.

[3] Gruber BD, Giehl RF, Friedel S, von Wirén N (2013). Plasticity of the *Arabidopsis* root system under nutrient deficiencies. Plant Physiol.

[4] Lynch JP, Chimungu JG, Brown KM (2014). Root anatomical phenes associated with water acquisition from drying soil: targets for crop improvement. J Exp Bot.

[5] Schmidt JE, Gaudin ACM (2017). Toward an integrated root ideotype for irrigated systems. Trends Plant Sci.

[6] Lasda E, Parker R (2014). Circular RNAs: diversity of form and function. RNA.

[7] Chen LL, Yang L (2015). Regulation of circRNA biogenesis. RNA Biol.

[8] Salzman J, Gawad C, Wang PL, Lacayo N, Brown PO (2012). Circular RNAs are the predominant transcript isoform from hundreds of human genes in diverse cell types. PLoS ONE.

[9] Jeck WR, Sharpless NE (2014). Detecting and characterizing circular RNAs. Nat Biotechnol.

[10] Westholm JO, Miura P, Olson S, Shenker S, Joseph B, Sanfilippo P, Celniker SE, Graveley BR, Lai EC (2014). Genome-wide analysis of *Drosophila* circular RNAs reveals their structural and sequence properties and age-dependent neural accumulation. Cell Rep..

[11] Fan XY, Zhang XN, Wu XL, Guo HS, Hu YQ, Tang FC, Huang YY (2015). Single-cell RNA-seq transcriptome analysis of linear and circular RNAs in mouse preimplantation embryos. Genome Biol.

[12] Sablok G, Zhao H, Sun X (2016). Plant circular RNAs (circRNAs): transcriptional regulation beyond miRNAs in plants. Mol Plant.

[13] Ye CY, Chen L, Liu C, Zhu QH, Fan L (2015). Widespread noncoding circular RNAs in plants. New Phytol.

[14] Pan T, Sun XQ, Liu YX, Li H, Deng GB, Lin HH, Wang SH (2018). Heat stress alters genome-wide profiles of circular RNAs in *Arabidopsis*. Plant Mol Biol.

[15] Liu TF, Zhang L, Chen G, Shi TL (2017). Identifying and characterizing the circular RNAs during the lifespan of *Arabidopsis* leaves. Front Plant Sci.

[16] Chen G, Cui JW, Wang L, Zhu YF, Lu ZG, Jin B (2017). Genome-wide identification of circular RNAs in *Arabidopsis thaliana*. Front Plant Sci.

[17] Zhao W, Cheng YH, Zhang C, You QB, Shen XJ, Guo W, Jiao YQ (2017). Genome-wide identification and characterization of circular RNAs by high throughput sequencing in soybean. Sci Rep.

[18] Lu TT, Cui LL, Zhou Y, Zhu CR, Fan DL, Gong H, Zhao Q, Zhou CC, Zhao Z, Lu DF (2015). Transcriptome-wide investigation of circular RNAs in rice. RNA.

[19] Zuo J, Wang Q, Zhu B, Luo Y, Gao L (2016). Deciphering the roles of circRNAs on chilling injury in tomato. Biochem Biophys Res Commun.

[20] Darbani B, Noeparvar S, Borg S (2016). Identification of circular RNAs from the parental genes involved in multiple aspects of cellular metabolism in barley. Front Plant Sci.

[21] Zhao T, Wang L, Li S, Xu M, Guan X, Zhou B (2017). Characterization of conserved circular RNA in polyploid Gossypium species and their ancestors. FEBS Lett.

[22] Chen L, Zhang P, Fan Y, Lu Q, Li Q, Yan J, Muehlbauer GJ, Schnable PS, Dai M, Li L (2017). Circular RNAs mediated by transposons are associated with transcriptomic and phenotypic variation in maize. New Phytol.

[23] Wang YX, Yang M, Wei SM, Qin FJ, Zhao HJ, Suo B (2017). Identification of circular RNAs and their targets in leaves of *Triticum aestivum* L. under dehydration stress. Front Plant Sci.

[24] Wang PL, Bao Y, Yee MC, Barrett SP, Hogan GJ, Olsen MN, Dinneny JR, Brown PO, Salzman J (2014). Circular RNA is expressed across the eukaryotic tree of life. PLoS ONE.

[25] Wang ZP, Liu YF, Li DW, Li L, Zhang Q, Wang SB, Huang HW (2017). Identification of circular RNAs in kiwifruit and their species-specific response to bacterial canker pathogen invasion. Front Plant Sci.

[26] Ren YZ, He X, Liu DC, Li JJ, Zhao XQ, Li B, Tong YP, Zhang AM, Li ZS (2012). Major quantitative trait loci for seminal root morphology of wheat seedlings. Mol Breed.

[27] Zadoks JC, Chang TT, Konzak CF (1974). A decimal code for the growth stages of cereals. Weed Res.

[28] Memczak S, Jens M, Elefsinioti A, Torti F, Krueger J, Rybak A, Maier L, Mackowiak SD, Gregersen LH, Munschauer M (2013). Circular RNAs are a large class of animal RNAs with regulatory potency. Nature.

[29] Anders S, Huber W (2010). Differential expression analysis for sequence count data. Genome Biol.

[30] Zhou L, Chen JH, Li ZZ, Li XX, Hu XD, Huang Y, Zhao XK, Liang CZ, Wang Y, Sun L (2010). Integrated profiling of microRNAs and mRNAs: microRNAs located on Xq27.3 associate with clear cell renal cell carcinoma. PLoS ONE.

[31] Dai X, Zhao PX (2011). psRNATarget: a plant small RNA target analysis server. Nucleic Acids Res.

[32] Young MD, Wakefeld MJ, Smyth GK, Oshlack A (2010). Gene ontology analysis for RNA-seq: accounting for selection bias. Genome Biol.

[33] Lynch JP (2013). Steep, cheap and deep: an ideotype to optimize water and N acquisition by maize root systems. Ann Bot.

[34] Ren YZ, Qian YY, Xu YH, Zou CQ, Liu DC, Zhao XQ, Zhang AM, Tong YP (2017). Characterization of QTLs for root traits of wheat grown under different nitrogen and phosphorus supply levels. Front Plant Sci.

[35] Hansen TB, Jensen TI, Clausen BH, Bramsen JB, Finsen B, Damgaard CK, Kjems J (2013). Natural RNA circles function as efficient microRNA sponges. Nature.

[36] Zhang S, Zhu D, Li H, Li H, Feng C, Zhang W (2017). Characterization of circRNA-associated-ceRNA networks in a senescence-accelerated mouse prone 8 brain. Mol Ther.

[37] Cai H, Lu Y, Xie W, Zhu T, Lian X (2012). Transcriptome response to nitrogen starvation in rice. J Biosci.

[38] Feng K, Nie X, Cui L, Deng P, Wang M, Song W (2017). Genome-wide identification and characterization of salinity stress-responsive miRNAs in wild emmer wheat (*Triticum turgidum* ssp. *dicoccoides*). Genes.

